# Utilization of Patient-Reported Outcomes to Guide Symptom Management during Stereotactic Body Radiation Therapy for Clinically Localized Prostate Cancer

**DOI:** 10.3389/fonc.2017.00227

**Published:** 2017-10-16

**Authors:** Malika Danner, Ming-yang Hung, Thomas M. Yung, Marilyn Ayoob, Siyuan Lei, Brian T. Collins, Simeng Suy, Sean P. Collins

**Affiliations:** ^1^Department of Radiation Medicine, MedStar Georgetown University Hospital, Washington, DC, United States

**Keywords:** prostate cancer, stereotactic body radiation therapy, CyberKnife, Expanded Prostate Cancer Index Composite, patient-reported outcome

## Abstract

**Introduction:**

Utilization of patient-reported outcomes (PROs) to guide symptom management during radiation therapy is increasing. This study focuses on the use of the Expanded Prostate Cancer Index Composite for Clinical Practice (EPIC-CP) as a tool to assess urinary and bowel bother during stereotactic body radiation therapy (SBRT) and its utility in guiding medical management.

**Methods:**

Between September 2015 and January 2017, 107 patients with clinically localized prostate cancer were treated with 35–36.25 Gy *via* SBRT in five fractions. PROs were assessed using EPIC-CP 1 h prior to the first fraction and after each subsequent fraction. Symptom management medications were prescribed based on the physician clinical judgment or if patients reported a moderate to big problem. Clinical significance was assessed using a minimally important difference of 1/2 SD from baseline score.

**Results:**

A median baseline EPIC-CP urinary symptom score of 1.5 significantly increased to 3.7 on the day of the final treatment (*p* < 0.0001). Prior to treatment, 9.3% of men felt that their overall urinary function was a moderate to big problem that increased to 28% by the end of the fifth treatment. A median baseline EPIC-CP bowel symptom score of 0.3 significantly increased to 1.4 on the day of the final treatment (*p* < 0.0001). Prior to treatment, 1.9% of men felt that their overall bowel function was a moderate to big problem that increased to 3.7% by the end of the fifth treatment. The percentage of patients requiring an increased dose of alpha-antagonist increased to 47% by the end of treatment, and an additional 28% of patients required a short steroid taper to manage moderate to big urinary problems. Similarly, the percentage of patients requiring antidiarrheals reached 12% by the fifth treatment.

**Conclusion:**

During the course of SBRT, an increasing percentage of patients experienced clinically significant symptoms many of which required medical management. Monitoring patient symptoms during treatment allowed for prompt detection and management of acute urinary and bowel symptoms. The usage of symptom management medications was high in this study compared to historical controls and may be due to increased physician awareness of moderate to big patient problems.

## Background

The utilization of stereotactic body radiation therapy (SBRT) for the treatment of clinically localized prostate cancer is increasing (1). SBRT precisely delivers high doses of radiation to the prostate while minimizing radiation exposure to the bladder and rectum. With SBRT, biochemical disease-free survival is high, while late morbidity has been comparable to conventionally fractionated radiation therapy (2, 3). Currently, there are limited data suggesting that any particular treatment for prostate cancer has superior outcomes compared to the others (4, 5). As a result, the patient’s choice of intervention is generally guided by their preference for a given treatment’s side effect profile. Prostate SBRT may have a higher rate of grade 2 acute toxicities than conventionally fractionated radiation therapy (6, 7). However, they may be of shorter duration, and many patients may still choose SBRT due to its convenience (8) and lower PSA nadirs (9). Thus, prostate SBRT utilization is likely to continue to gain popularity emphasizing the importance of developing clinical strategies to identify urinary and bowel symptoms early and individualize management to improve each patient’s quality of life (QOL) during treatment.

Utilization of patient-reported outcomes (PROs) to guide acute symptom management during radiation therapy is increasing (10). One such example is the Expanded Prostate Cancer Index Composite-26 (EPIC-26), which is the standard questionnaire employed to assess treatment-related morbidity on prostate cancer clinical trials (11–13). However, its 26 questions may be too burdensome for repetitive measures over a short-time period. Excessive respondent burden has shown to reduce questionnaire response rates and data accuracy (14). The Expanded Prostate Cancer Index Composite for Clinical Practice (EPIC-CP) is a single-page version of the EPIC-26 that was developed for general clinical use (15, 16). It is a psychometrically validated questionnaire that allows for multiple assessments over a short period of time while minimizing patient burden (15). It contains 16 questions that assess several health-related QOL domains including the urinary and bowel domains.

Limited data are available on PROs during SBRT. Urinary and bowel symptoms commonly present during the 2-week course of SBRT. Further knowledge in this area would improve patient expectations prior to treatment. The objective of this study was to prospectively collect and report the urinary and bowel QOL outcomes during SBRT in patients with clinically localized prostate cancer. In addition, we demonstrate the feasibility of incorporating the EPIC-CP into daily clinical practice during a course of radiation therapy and its utilization in symptom management.

## Methods

### Patient Selection

The Medstar Georgetown University Hospital Internal Review Board (IRB) approved this single-institution prospective QOL study (IRB 12-1175). Patients eligible for study inclusion had prostate cancer treated with SBRT over 2 weeks. All patients provided informed consent prior to treatment. Patient and treatment characteristics such as age, race, prostate volume, pretreatment PSA, T stage, Gleason score, risk groups, hormone treatment, and dose were acquired from the medical records. Risk groups were defined using the D’Amico criteria.

### SBRT Treatment Planning and Delivery

Stereotactic body radiation therapy treatment planning and delivery were performed as previously described (6, 7). Gold fiducials were placed into the prostate using ultrasound guidance. Fused thin cut CT images and high-resolution MR images were used for treatment planning. The clinical target volume (CTV) included the prostate and proximal seminal vesicles. The planning target volume (PTV) included a 3-mm (inferior, superior, and posterior) or 5-mm (anterolateral) expansion around the CTV. A prescription dose of 35–36.25 Gy was delivered to the PTV in five fractions of 7–7.25 Gy over 2 weeks. The bladder, prostatic urethra, membranous urethra, and rectum were contoured and evaluated with dose–volume histogram analysis during treatment planning as previously described (7). Target position was confirmed multiple times during each treatment with a minimum of three properly placed fiducials (17). Prophylactic alpha-adrenergic antagonists were initiated 5 days prior to SBRT and continued throughout the treatment course. Patients were instructed to start a low-residual diet 5 days prior to SBRT simulation and remain on it until 1 week after the completion of treatment. Enemas were performed prior to simulation and each treatment. Symptom management medications were prescribed based on physician clinical judgment (SPC) or if patients reported a moderate to big problem. In general, patients with bothersome urinary symptoms were managed with alpha-adrenergic antagonist dose increases. Patients with refractory urinary symptoms were treated with a short steroid taper (4 mg dexamethasone for 7 days followed by 2 mg dexamethasone for 7 days). Patients with bothersome bowel symptoms were managed with anti-diarrheal medication (loperamide).

### Follow-up and Statistical Analysis

On-treatment PRO assessment time points were prospectively collected 1 h prior to the first SBRT fraction and after each subsequent treatment using the EPIC-CP as part of the on-treatment visit (OTV). The EPIC-CP is a validated tool that measures urinary and bowel bother (15). Furthermore, EPIC-CP scores at initial consultation (baseline) and 1 week post-SBRT were obtained from the EPIC-26 questionnaire that is filled out at these time points in our clinic (6, 7). The score for each EPIC-CP question was calculated from the corresponding question in the EPIC-26 as previously described (18). To statistically compare changes between time points, the levels of responses were assigned a score, and the significance of the mean changes in the scores was assessed by paired *t*-test. Each domain contained three questions that were assigned numerical scores of 0–4. The domain scores were the summation of the three question scores (0–12) with higher numbers corresponding to increased bother. The responses to individual questions were grouped into three clinically relevant categories (no problem, very small to small problem, and moderate to big problem). Wilcoxon signed-rank test and chi-square analysis were used to assess differences in QOL scores in comparison to baseline. Paired *t*-test was used to assess the significance of the change in scores. Clinical significance was assessed using minimally important difference (MID) in EPIC-CP score, which is defined as a difference of one half SD from baseline score (19).

## Results

**Figure 1:**
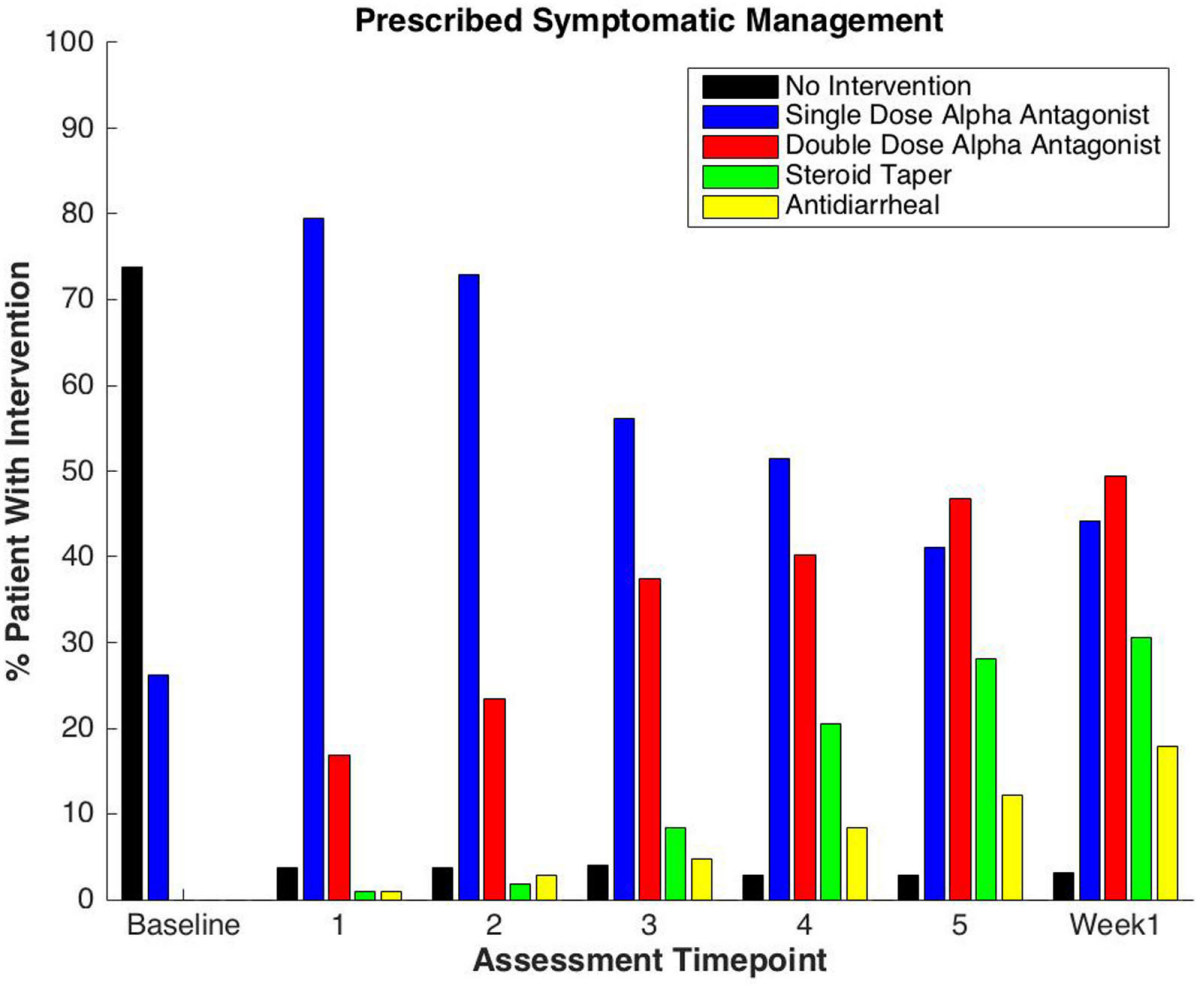
Percentage of patients prescribed specific symptom management medications during prostate stereotactic body radiation therapy.

One-hundred seven patients with clinically localized prostate cancer were treated on a prospectively conducted IRB-approved institutional protocol between September 2015 and January 2017. The median patient age was 71 years (53–86 years) (Table 1). 66.4% patients were white and 29.9% were black. 11.2% patients were low risk, 80.4% patients were intermediate risk, and 8.4% patients were high risk. The median prostate volume was 37.4 cc (12.5–113 cc). 93.5% of patients were treated with 36.25 Gy in five 7.25 Gy fractions (Table 1). 96% of the patients were taking alpha-adrenergic antagonists prior to receiving the first SBRT treatment (Table 2; Figure 1).

**Table 1:** Patient baseline characteristics and treatment specifics.

Patients (*N* = 107)		%	*n*
Age (years), median (range)	71 (53–86)		
	<60	2.8	3
	60–69	39.3	42
	70–79	51.4	55
	≥80	6.5	7
Race	White	66.4	71
	Black	29.9	32
	Other	3.7	4
Prostate volume (cc), median (range)	37.4 (12.5–113)		
Pre-Tx PSA (ng/ml), median (range)	6.4 (0.0–40.8)		
T stage	T1c	60.7	65
	T2a	15.9	17
	T2b	20.6	22
	T2c	2.8	3
	T3	0	0
Gleason score	3 + 3 = 6	29.0	31
	3 + 4 = 7	40.2	43
	4 + 3 = 7	23.4	25
	4 + 4 = 8	4.7	5
	4 + 5 = 9	2.8	3
Risk groups	Low	11.2	12
	Intermediate	80.4	86
	High	8.4	9
Hormone treatment	Yes	22.4	24
	No	77.6	83
Dose (Gy)	35	6.5	7
	36.25	93.5	100

**Table 2:** Percentage of patients prescribed specific symptom management medications during prostate stereotactic body radiation therapy.

Drug used	Baseline	Treatment					1 week
		1	2	3	4	5	
None	73.8	3.7	3.7	4	2.8	2.8	3.2
Alpha-antagonist 1 dose	26.2	79.4	72.9	56.1	51.4	41.1	44.2
Alpha-antagonist 2 dose	0.0	16.8	23.4	37.4	40.2	46.7	49.5
Steroid	0.0	0.9	1.9	8.4	20.6	28.0	30.5
Antidiarrheal	0.0	0.9	2.8	4.7	8.4	12.1	17.9

**Figure 2:**
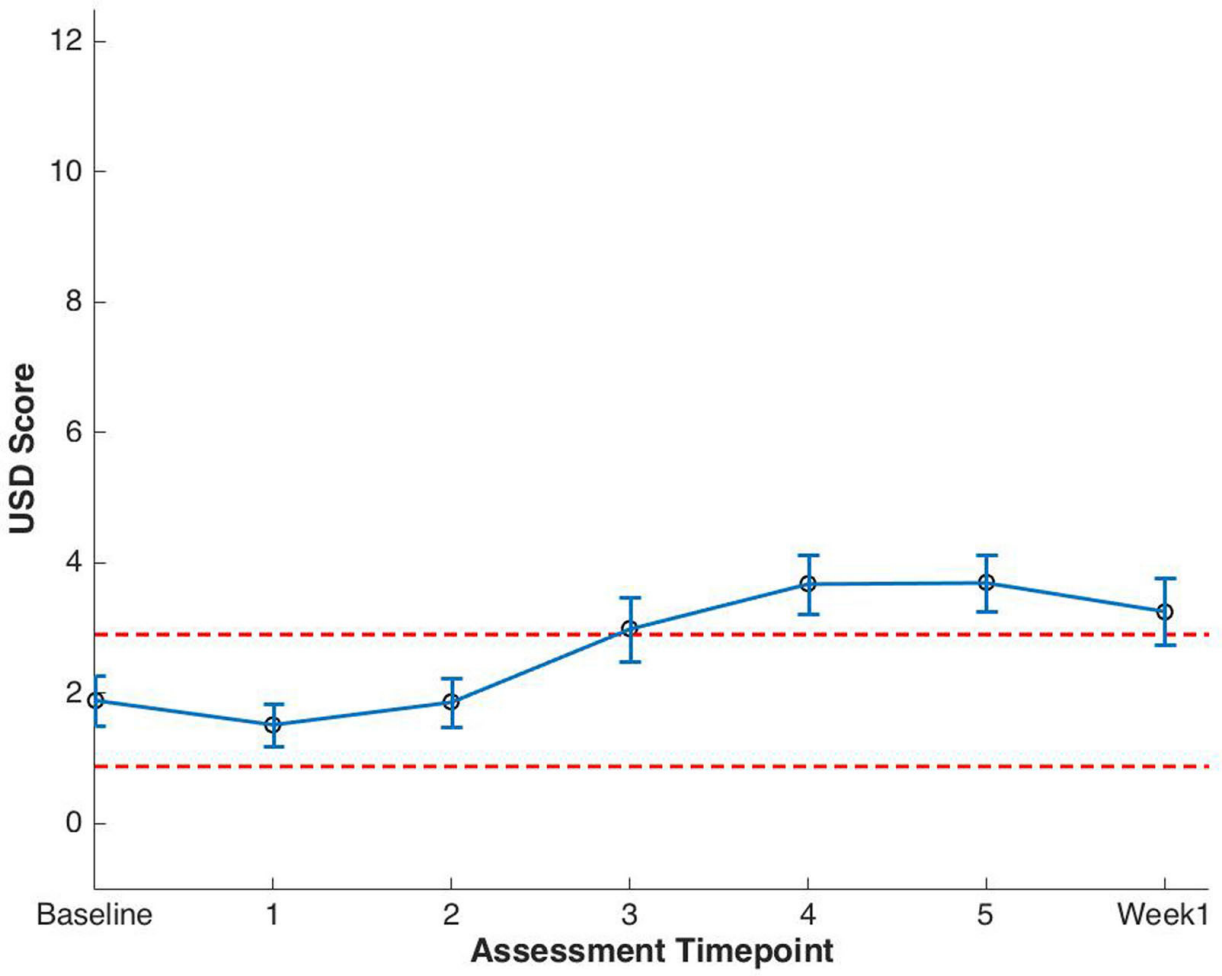
Expanded Prostate Cancer Index Composite for Clinical Practice (EPIC-CP) urinary summary domain (USD) scores at baseline and during stereotactic body radiation therapy for prostate cancer. Thresholds for clinically significant changes in scores (1/2 SD above and below the baseline) are marked with dashed lines. EPIC-CP scores range from 0 to 12 with higher values representing poorer health-related quality of life.

Baseline EPIC-CP summary domain scores and mean changes from baseline to week 1 post-SBRT are shown in Table 3. A median baseline EPIC-CP urinary symptom score of 1.9 significantly increased to 3.3 1 week after the completion of treatment (*p* < 0.0001) (Table 3; Figure 2). Changes in EPIC-CP urinary symptom score were clinically significant by third treatment, exceeding the MID of 1.0. Prior to treatment, 11.2% of men felt that their overall urinary function was a moderate to big problem that increased to 37.9% by 1 week after the completion of treatment (Table 4). The proportion of patients experiencing clinically meaningful specific urinary symptoms (moderate to big problem) also increased: dysuria from 0 (baseline) to 15.8% (1 week post-SBRT); urine retention from 6.5 (baseline) to 11.6% (1 week post-SBRT); and urinary frequency from 11.2 (baseline) to 30.5% (1 week post-SBRT) (Table 4). The percentage of patients requiring an increased dose of alpha-antagonist increased to 47% by the fifth treatment, and an additional 28% of patients required a short steroid taper to manage moderate to big urinary problems (Table 2; Figure 1).

**Table 3:** Changes in Expanded Prostate Cancer Index Composite for Clinical Practice urinary and bowel domain scores during sterotactic body radiation therapy for prostate cancer.

	Urination			Bowel		
	Mean score	CI	*p*	Mean score	CI	*p*
Baseline	1.9	0.4	NA	0.8	0.3	NA
Treatment 1	1.5	0.3	0.0163*	0.3	0.2	0.0001*
Treatment 2	1.9	0.4	0.7453	0.5	0.2	0.0293*
Treatment 3	3.0	0.5	0.0000*	0.8	0.3	0.9826
Treatment 4	3.7	0.5	0.0000*	1.3	0.4	0.0315*
Treatment 5	3.7	0.4	0.0000*	1.4	0.4	0.0145*
Week 1	3.3	0.5	0.0000*	3.4	0.7	0.0000*

*Statistical significance (α = 0.05).

**Table 4:** Patient-reported responses to Expanded Prostate Cancer Index Composite for Clinical Practice questions.

	Baseline	Treatment					1 week
		1	2	3	4	5	
Number (*n*)	93	107	107	107	107	107	95
**Dysuria**							
No problem (%)	94.4	97.2	86.0	59.8	42.1	41.1	50.5
Very small to small problem (%)	5.6	1.9	13.1	34.6	47.7	47.7	33.7
Moderate to big problem (%)	0.0	0.9	0.9	5.6	10.3	11.2	15.8
*p-value*		0.4844	0.0510	0.0000*	0.0000*	0.0000*	0.0000*
**Urinary retention**							
No problem (%)	60.7	69.2	57.9	42.1	35.5	31.8	63.2
Very small to small problem (%)	32.7	28.0	36.4	45.8	56.1	59.8	25.3
Moderate to big problem (%)	6.5	2.8	5.6	12.1	8.4	8.4	11.6
*p-value*		0.0163	0.9292	0.0001*	0.0000*	0.0001*	0.8920
**Urinary frequency**							
No problem (%)	36.4	43.0	40.2	31.8	18.7	19.6	30.5
Very small to small problem (%)	52.3	49.5	52.3	52.3	63.6	64.5	38.9
Moderate to big problem (%)	11.2	7.5	7.5	15.9	17.8	15.9	30.5
*p-value*		0.0743	0.0994	0.4035	0.0014*	0.0010*	0.0241*
**Overall urinary problem**							
No problem (%)	31.8	39.3	27.1	16.8	9.3	8.4	15.8
Very small to small problem (%)	57.0	51.4	63.6	62.6	66.4	63.6	46.3
Moderate to big problem (%)	11.2	9.3	9.3	20.6	24.3	28.0	37.9
*p-value*		0.1195	0.7589	0.0021*	0.0000*	0.0000*	0.0002*
**Bowel urgency/rectal pain**							
No problem (%)	73.8	94.4	89.7	79.4	76.6	68.2	37.9
Very small to small problem (%)	24.3	4.7	10.3	18.7	18.7	27.1	38.9
Moderate to big problem (%)	1.9	0.9	0.0	1.9	4.7	4.7	23.2
*p-value*		0.0000*	0.0005*	0.1472	0.8773	0.5002	0.0000*
**Bowel frequency**							
No problem (%)	90.7	96.3	90.7	80.4	69.2	72.9	67.4
Very small to small problem (%)	7.5	2.8	7.5	19.6	26.2	21.5	17.9
Moderate to big problem (%)	1.9	0.9	1.9	0.0	4.7	5.6	14.7
*p-value*		0.1562	0.6703	0.0969	0.0003*	0.0004*	0.0000*
**Overall bowel problem**							
No problem (%)	81.3	93.5	87.9	83.2	77.6	71.0	41.1
Very small to small problem (%)	15.0	4.7	11.2	15.9	16.8	25.2	37.9
Moderate to big problem (%)	3.7	1.9	0.9	0.9	5.6	3.7	21.1
*p-value*		0.0112*	0.1136	0.6455	0.3372	0.0750	0.0000*

*Statistical significance (α = 0.05).

**Figure 3:**
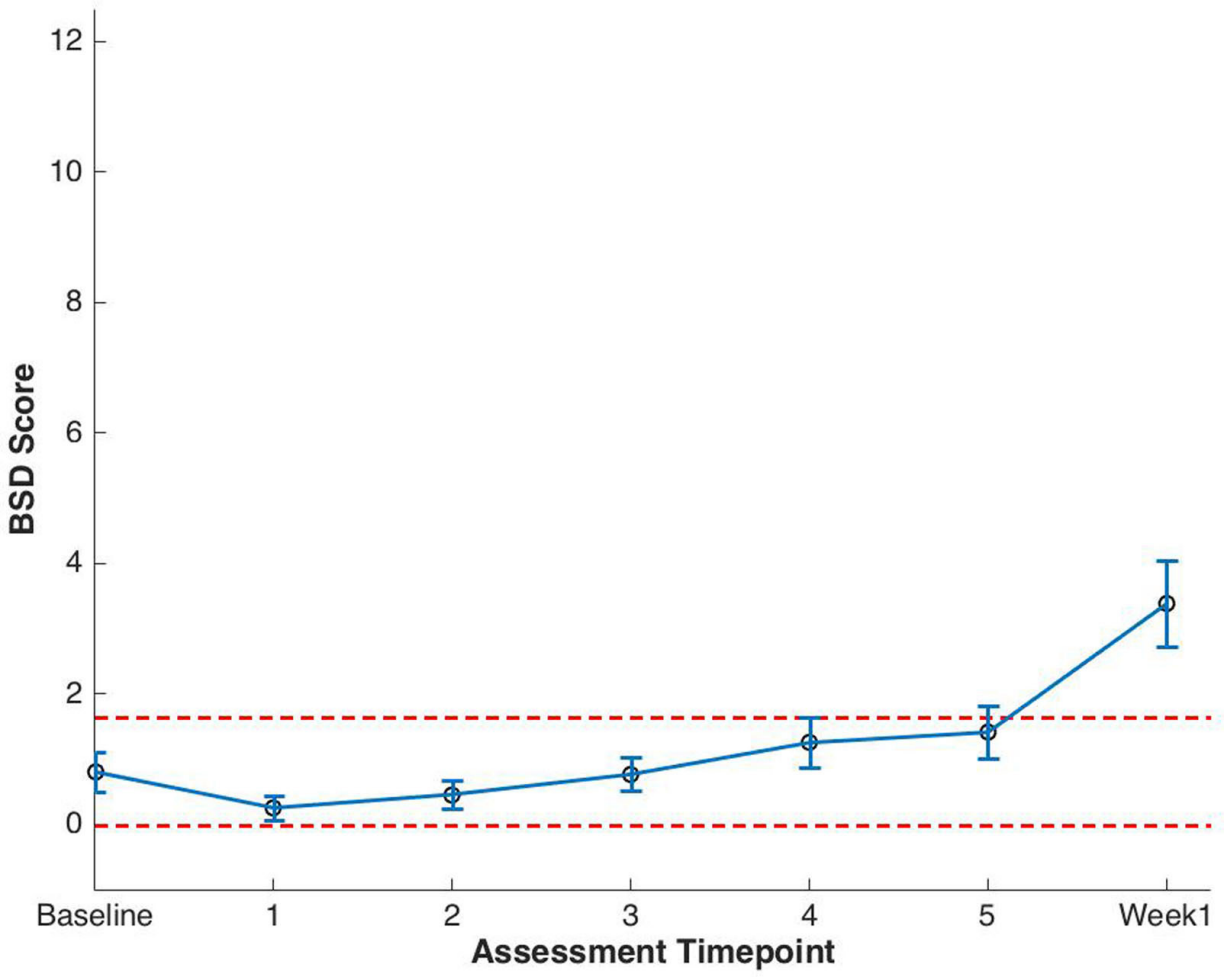
Expanded Prostate Cancer Index Composite for Clinical Practice (EPIC-CP) bowel summary domain (BSD) scores at baseline and during stereotactic body radiation therapy for prostate cancer. Thresholds for clinically significant changes in scores (1/2 SD above and below the baseline) are marked with dashed lines. EPIC-CP scores range from 0 to 12 with higher values representing poorer health-related quality of life.

Clinically significant bowel symptoms were less common prior to and during SBRT treatment than urinary symptoms. A median baseline EPIC-CP bowel symptom score of 0.8 significantly increased to 3.4 1 week after the completion of treatment (*p* < 0.0001) (Table 3; Figure 3). Changes in EPIC-CP bowel symptom score were clinically significant by 1 week after the completion of treatment, exceeding the MID of 0.8. At baseline, 3.7% of men felt that their overall bowel function was a moderate to big problem that increased to 21.1% 1 week after the completion of treatment (Table 4). The proportion of patients experiencing clinically meaningful specific bowel symptoms (moderate to big problem) also increased: bowel urgency/rectal pain from 1.9% (baseline) to 23.2% (1 week post-SBRT) and bowel frequency from 1.9% (baseline) to 14.7% (1 week post-SBRT) (Table 4). The percentage of patients requiring antidiarrheals reached 12% by the fifth treatment (Table 2; Figure 1).

## Discussion

Acute side effects are an important consideration when prostate cancer patients are deciding on their treatment. SBRT is an extremely hypofractionated form of radiation therapy that may have more severe acute side effects than conventionally fractionated radiation therapy (6, 7). A better understanding of the acute side effect profile of prostate SBRT would facilitate better patient education. Understanding the risk of potential acute side effects prior to beginning treatment and appropriately managing them when they occur may also prevent treatment regret (8). Thus, this study assessed patient-reported urinary and bowel symptoms during SBRT.

In this study, we assessed urinary and bowel symptoms on every day of treatment using the EPIC-CP. The EPIC-CP is a one-page questionnaire that was completed by the patient with the aid of the study coordinator (Malika Danner). Due to the low response burden, we were able to obtain a high response rate (100%) on every day of treatment. In the opinion of the radiation oncologist (SC), the questionnaire was helpful in guiding management decisions and did not greatly increase the length of the OTV.

Treatment interruptions during radiotherapy due to acute urinary and bowel symptoms may negatively affect cancer control (20). During the 2-week course of SBRT treatment, 46% of patients developed moderate to big problems with urinary and/or bowel function. This appears greater than the number of patients who experienced clinically significant symptoms during conventionally fractionated radiation therapy (21–23). Although the incidence of acute grade 2 toxicity appears high in this study, no patient’s treatment was prolonged due to treatment related symptoms. This is possibly due to our timely medical management of moderate to big problems.

The usage of symptom management medications was high in this study as compared to historical controls (24). In this study, approximately 50% of patients required acute medical management for clinically significant urinary symptoms. This is higher than the 35% of patient whom we first reported who required medical management of urinary symptoms in the first month following prostate SBRT (24). Likewise, in this study, 12% of patients required acute medical management for clinically significant bowel symptoms. Once again, this is higher than the 5% of men who required medical management of bowel symptoms in our prior research (24). In the opinions of the authors, this increased rate of medication prescription may be due to increased physician awareness of moderate to big patient problems secondary to the use of frequent questionnaire administration.

This study has several limitations. Although the EPIC-CP has been validated to assess QOL outcomes in prostate cancer patients treated with radiation therapy (15), it has not specifically been validated to show changes in urinary and bowel symptoms on an every-other-day basis (25). EPIC-CP questionnaires were not administered at baseline and 1 week posttreatment. Thus, to obtain EPIC-CP scores at these time points for comparison, the answers to the corresponding EPIC-26 question were utilized to generate an EPIC-CP score at baseline and 1-week post-SBRT. In addition, the EPIC-CP does not have a question regarding rectal bleeding (18). However, rectal bleeding is an uncommon acute bowel symptom following SBRT (<5%) (6) and would likely be captured in the overall bowel problem question. Most importantly, we had difficulty comparing our results with those of conventionally fractionated radiation therapy due to the utilization of alternative questionnaires with different definitions of clinically significant symptoms. The recent definition of a standard set of patient-centered outcomes for men with localized prostate cancer may decrease this problem in future studies (11).

## Conclusion

During the course of SBRT, an increasing percentage of patients experienced clinically significant symptoms many of which required medical management. Monitoring patient symptoms during treatment allowed for prompt detection and management of acute urinary and bowel symptoms. The usage of symptom management medications was high in this study compared to historical controls and may be due to increased physician awareness of moderate to big patient problems.

## Ethics Statement

The Medstar Georgetown University Hospital Institutional Review Board (IRB) approved this single-institution prospective quality of life study (IRB 12-1175).

## Author Contributions

MD and M-yH are lead authors who participated in data collection, manuscript drafting, table/figure creation, and manuscript revision. MA and TY aided in data collection. SL is the dosimetrist who contributed dosimetric data and figures. BC and SS are senior authors who aided in drafting the manuscript and manuscript revision. SC is the corresponding author who initially developed the concept and drafted and revised the manuscript. All authors read and approved the final manuscript.

## Conflict of Interest Statement

SC and BC serve as clinical consultants to Accuray Inc. The Department of Radiation Medicine at Georgetown University Hospital receives a grant from Accuray to support a research coordinator. The other authors declare that they have no competing interests.

## Abbreviations

ADT, androgen deprivation therapy; BSD, bowel summary domain; CTV, clinical target volume; DVH, dose–volume histogram; EBRT, external beam radiation therapy; EPIC, Expanded Prostate Index Composite; GTV, gross target volume; HRQOL, health-related quality of life; MID, minimally important difference; OTV, on-treatment visit; PTV, planning target volume; QOL, quality of life; SBRT, stereotactic body radiation therapy; USD, urinary summary domain.

## References

[1] Baker BR Basak R Mohiuddin JJ Chen RC. Use of stereotactic body radiotherapy for prostate cancer in the United States from 2004 through 2012. Cancer (2016) 122(14):2234–41. 10.1002/cncr.30034 27171855

[2] King CR Collins S Fuller D Wang PC Kupelian P Steinberg M Health-related quality of life after stereotactic body radiation therapy for localized prostate cancer: results from a multi-institutional consortium of prospective trials. Int J Radiat Oncol Biol Phys (2013) 87(5):939–45. 10.1016/j.ijrobp.2013.08.019 24119836

[3] King CR Freeman D Kaplan I Fuller D Bolzicco G Collins S Stereotactic body radiotherapy for localized prostate cancer: pooled analysis from a multi-institutional consortium of prospective phase II trials. Radiother Oncol (2013) 109(2):217–21. 10.1016/j.radonc.2013.08.030 24060175

[4] Donovan JL Hamdy FC Lane JA Mason M Metcalfe C Walsh E Patient-reported outcomes after monitoring, surgery, or radiotherapy for prostate cancer. N Engl J Med (2016) 375(15):1425–37. 10.1056/NEJMoa1606221 27626365 PMC5134995

[5] Hamdy FC Donovan JL Lane JA Mason M Metcalfe C Holding P 10-year outcomes after monitoring, surgery, or radiotherapy for localized prostate cancer. N Engl J Med (2016) 375(15):1415–24. 10.1056/NEJMoa1606220 27626136

[6] Paydar I Cyr RA Yung TM Lei S Collins BT Chen LN Proctitis 1 week after stereotactic body radiation therapy for prostate cancer: implications for clinical trial design. Front Oncol (2016) 6:167. 10.3389/fonc.2016.00167 27489794 PMC4951492

[7] Repka MC Guleria S Cyr RA Yung TM Koneru H Chen LN Acute urinary morbidity following stereotactic body radiation therapy for prostate cancer with prophylactic alpha-adrenergic antagonist and urethral dose reduction. Front Oncol (2016) 6:122. 10.3389/fonc.2016.00122 27242962 PMC4870496

[8] Shaverdian N Verruttipong D Wang PC Kishan AU Demanes DJ McCloskey S Exploring value from the patient’s perspective between modern radiation therapy modalities for localized prostate cancer. Int J Radiat Oncol Biol Phys (2017) 97(3):516–25. 10.1016/j.ijrobp.2016.11.007 28126301

[9] Kishan AU Wang PC Upadhyaya SK Hauswald H Demanes DJ Nickols NG SBRT and HDR brachytherapy produce lower PSA nadirs and different PSA decay patterns than conventionally fractionated IMRT in patients with low- or intermediate-risk prostate cancer. Pract Radiat Oncol (2016) 6(4):268–75. 10.1016/j.prro.2015.11.002 26850649

[10] Basch E Deal AM Kris MG Scher HI Hudis CA Sabbatini P Symptom monitoring with patient-reported outcomes during routine cancer treatment: a randomized controlled trial. J Clin Oncol (2016) 34(6):557–65. 10.1200/JCO.2015.63.0830 26644527 PMC4872028

[11] Martin NE Massey L Stowell C Bangma C Briganti A Bill-Axelson A Defining a standard set of patient-centered outcomes for men with localized prostate cancer. Eur Urol (2015) 67(3):460–7. 10.1016/j.eururo.2014.08.075 25234359

[12] Sanda MG Dunn RL Michalski J Sandler HM Northouse L Hembroff L Quality of life and satisfaction with outcome among prostate-cancer survivors. N Engl J Med (2008) 358(12):1250–61. 10.1056/NEJMoa074311 18354103

[13] Wei JT Dunn RL Litwin MS Sandler HM Sanda MG. Development and validation of the expanded prostate cancer index composite (EPIC) for comprehensive assessment of health-related quality of life in men with prostate cancer. Urology (2000) 56(6):899–905. 10.1016/S0090-4295(00)00858-X 11113727

[14] Rolstad S Adler J Ryden A. Response burden and questionnaire length: is shorter better? A review and meta-analysis. Value Health (2011) 14(8):1101–8. 10.1016/j.jval.2011.06.003 22152180

[15] Chang P Szymanski KM Dunn RL Chipman JJ Litwin MS Nguyen PL Expanded prostate cancer index composite for clinical practice: development and validation of a practical health related quality of life instrument for use in the routine clinical care of patients with prostate cancer. J Urol (2011) 186(3):865–72. 10.1016/j.juro.2011.04.085 21788038 PMC3807735

[16] Chipman JJ Sanda MG Dunn RL Wei JT Litwin MS Crociani CM Measuring and predicting prostate cancer related quality of life changes using EPIC for clinical practice. J Urol (2014) 191(3):638–45. 10.1016/j.juro.2013.09.040 24076307 PMC5006995

[17] Xie Y Djajaputra D King CR Hossain S Ma L Xing L. Intrafractional motion of the prostate during hypofractionated radiotherapy. Int J Radiat Oncol Biol Phys (2008) 72(1):236–46. 10.1016/j.ijrobp.2008.04.051 18722274 PMC2725181

[18] Lee JY Daignault-Newton S Heath G Scarlett S Sanda MG Chang P Multinational prospective study of patient-reported outcomes after prostate radiation therapy: detailed assessment of rectal bleeding. Int J Radiat Oncol Biol Phys (2016) 96(4):770–7. 10.1016/j.ijrobp.2016.07.038 27663760

[19] Norman GR Sloan JA Wyrwich KW. Interpretation of changes in health-related quality of life: the remarkable universality of half a standard deviation. Med Care (2003) 41(5):582–92. 10.1097/00005650-200305000-00007 12719681

[20] D’Ambrosio DJ Li T Horwitz EM Chen DY Pollack A Buyyounouski MK. Does treatment duration affect outcome after radiotherapy for prostate cancer? Int J Radiat Oncol Biol Phys (2008) 72(5):1402–7. 10.1016/j.ijrobp.2008.03.011 18472368 PMC2763099

[21] Chen RC Zhang Y Chen MH McMahon E Loffredo M McPherson CP Patient-reported quality of life during radiation treatment for localized prostate cancer: results from a prospective phase II trial. BJU Int (2012) 110(11):1690–5. 10.1111/j.1464-410X.2012.11117.x 22502770

[22] Diao K Lobos EA Yirmibesoglu E Basak R Hendrix LH Barbosa B Patient-reported quality of life during definitive and postprostatectomy image-guided radiation therapy for prostate cancer. Pract Radiat Oncol (2017) 7(2):e117–24. 10.1016/j.prro.2016.08.004 28274402

[23] Pinkawa M Fischedick K Asadpour B Gagel B Piroth MD Eble MJ Low-grade toxicity after conformal radiation therapy for prostate cancer – impact of bladder volume. Int J Radiat Oncol Biol Phys (2006) 64(3):835–41. 10.1016/j.ijrobp.2005.09.003 16289911

[24] Chen LN Suy S Uhm S Oermann EK Ju AW Chen V Stereotactic body radiation therapy (SBRT) for clinically localized prostate cancer: the Georgetown University experience. Radiat Oncol (2013) 8:58. 10.1186/1748-717X-8-58 23497695 PMC3610192

[25] Dowrick AS Wootten AC Murphy DG Costello AJ “We Used a Validated Questionnaire”: what does this mean and is it an accurate statement in urologic research? Urology (2015) 85(6):1304–10. 10.1016/j.urology.2015.01.046 25881867

